# A medical nutriment has supportive value in the treatment of colorectal cancer

**DOI:** 10.1038/sj.bjc.6601153

**Published:** 2003-07-29

**Authors:** F Jakab, Y Shoenfeld, Á Balogh, M Nichelatti, A Hoffmann, Zs Kahán, K Lapis, Á Mayer, P Sápy, F Szentpétery, A Telekes, L Thurzó, A Vágvölgyi, M Hidvégi

**Affiliations:** 1 Department of Surgery and Vascular Surgery, Uzsoki Teaching Hospital of Budapest, Hungary; 2 Department of Medicine ‘B’, Sackler Faculty of Medicine, Tel-Aviv University, Sheba Medical Center, Tel-Hashomer 52621, Israel; 3 Clinic of Surgery, University of Szeged, Hungary; 4 Biostatistics Unit, Associazione Malattie del Sangue, Hospital Niguarda Cà Granda, Milan, Italy; 5 Biromedicina First Hungarian Corporation for Cancer Research and Oncology, Budapest, Hungary; 6 Clinic of Oncotherapy, University of Szeged, Hungary; 7 1st Institute of Pathology and Experimental Cancer Research, Semmelweis University, Budapest, Hungary; 8 Budapest Center of Onco-Radiology, Uzsoki Teaching Hospital of Budapest, Hungary; 9 2nd Clinic of Surgery, University of Debrecen, Hungary; 10 Jewish University, Budapest, Hungary

**Keywords:** fermented wheat germ extract, colorectal cancer, cohort study, progression-free and overall survival

## Abstract

MSC (Avemar) is a medical nutriment of which preclinical and observational clinical studies suggested an antimetastatic activity with no toxicity. This open-label cohort trial has compared anticancer treatments plus MSC (9 g once daily) *vs* anticancer treatments alone in colorectal patients, enrolled from three oncosurgical centres; cohort allocation was on the basis of patients' choice. Sixty-six colorectal cancer patients received MSC supplement for more than 6 months and 104 patients served as controls (anticancer therapies alone): no statistical difference was noted in the time from diagnosis to the last visit between the two groups. End-point analysis revealed that progression-related events were significantly less frequent in the MSC group (new recurrences: 3.0 *vs* 17.3%, *P*<0.01; new metastases: 7.6 *vs* 23.1%, *P*<0.01; deaths: 12.1 *vs* 31.7%, *P*<0.01). Survival analysis showed significant improvements in the MSC group regarding progression-free (*P*=0.0184) and overall survivals (*P*=0.0278) probabilities. Survival predictors in Cox's proportional hazards were UICC stage and MSC treatment. Continuous supplementation of anticancer therapies with MSC for more than 6 months is beneficial to patients with colorectal cancer in terms of overall and progression-free survival.

A fermented wheat germ extract (code name: MSC; brand name: Avemar), standardised to methoxy-substituted benzoquinones and registered in Hungary in the year 2002 as medical nutriment (reg. no. 503), has recently been shown to induce apoptosis, to inhibit carbon flow to nucleic acid synthesis (Boros *et al*, 2001; Comín-Anduix *et al*, 2002) and to induce major histocompatibility complex (MHC) class I proteins' downregulation (Fajka-Boja *et al*, 2002) in tumour cells. It has also been published that the extract strongly inhibited the development of azoxymethane-induced colon cancer in rats (Zalatnai *et al*, 2001). In mice experiments, furthermore, the combination of 5-fluorouracil (5FU) injection plus the orally applied MSC was superior to 5FU alone in the inhibition of liver metastases formation of colon cancer origin (Hidvégi *et al*, 1999). An open-label, multicentre cohort study was therefore initiated to estimate the expected difference between the progression-free survivals of colorectal cancer patients receiving anticancer treatments alone or anticancer treatments supplemented with MSC.

## PATIENTS AND METHODS

An open-label comparative cohort trial has been conducted to determine if the addition of MSC to surgery and radio- and/or chemotherapy adds any therapeutic benefit compared to surgery and radio- and/or chemotherapy alone.

To be eligible, patients had to have cytologically or histologically documented colorectal cancer (Dukes A–D); a WHO performance status of 0, 1 or 2; adequate organ functions; life expectation of at least 6 months; and minimal age of 18 years. No restrictions were made regarding the date of the diagnosis. Similarly, no restrictions were made for prior radio- and/or chemotherapy, but all the patients had to undergo curative surgery at the time of diagnosis of their disease. This included complete removal of the primary tumour with adequate lymphadenectomy. In cases of existing distant metastatic lesions, indicated by preoperative investigations, hepatic and/or lung resection had also been carried out. At the time of the enrolment into this study, however, several patients had already had measurable and inoperable metastatic disease. Exclusion criteria were severe parenchymatous liver disease not connected to the metastatic cancer; severe kidney failure; respiratory insufficiency; history of severe cardiovascular disease; history of other type of cancer; pregnancy; lactation. The Regional Helsinki Committees approved the protocol, and all patients gave written informed consent before entering into the study.

The two cohorts of patients (MSC and ‘control’) were formed according to the patients' preference, since MSC was freely available as an over-the-counter product. Patients were asked if they prefer to take MSC, and thus enrolled in the MSC cohort. Those patients who refused to take the preparation formed the control cohort. The decision of treatment group assignment according to patients' preference was carried out on ethical basis, that is, those patients who otherwise would take MSC, got it free, and those who otherwise would not wish to take, served as control. By this procedure, no direct randomisation or stratification was performed. At the time of check-ups, control patients were always routinely asked if they take any dietary supplement or medical nutriment including MSC and, randomly chosen sera of these patients were analysed for 2-methoxy-benzoquinone (MBQ) and 2,6-dimethoxy-benzoquinone (DMBQ) contents, which are specific indicators of MSC administration. Patients answering ‘yes’ to the question of taking MSC, or having benzoquinone-positive sera, were excluded from the control cohort. The measurement of absorption of methoxy-substituted benzoquinones (MBQ and DMBQ), whose markers were in the form of glycosides in crude wheat germ, and were liberated as aglycones by the process of fermentation, served as a specific monitoring technique of MSC administration, and thus also of compliance. Sample preparation was carried out by solid-phase extraction of the sera, followed by reversed-phase high-performance liquid chromatography. Markers MBQ and DMBQ were detected by mass spectrometry using single-ion recording. The compounds were analysed by atmospheric pressure chemical ionisation. The detection limit was 2 ng per 10 ml serum. Besides this analytical monitoring, patients of the MSC cohort had to account for the empty containers of the nutriment before having received the new supply.

The survival analysis was chosen with power 1–*β*=0.8=80% and with a significance level *α*=0.05=5%; for these options, using the Freedman's procedure, the minimal required sample size was 62 patients per group, assuming a disease-free survival probability in the control group to be roughly equal to 50% at average follow-up, and an estimated disease-free probability increased by 25% in the MSC group, as deducible from previous animal studies, as well as from early clinical experiences.

Beyond the anticancer therapy for the group of patients of the MSC cohort, 9 g of MSC, dissolved in 150 ml of water, were taken orally once daily uninterruptedly and continuously throughout the study but, at least for more than 6 months. The treatment time was measured as the interval between the first and the last visits completed. Patients of the control cohort received the anticancer treatments alone. The term ‘anticancer treatments’ stands for the 5-FU-based standard Mayo Clinic chemotherapy regimen and/or radiation therapy following surgery.

All patients were evaluated at baseline, at the end of the first month, and every 12 weeks afterwards. Evaluation included assessment of all measurable lesions by imaging (radiographic, ultrasonic or magnetic resonance) techniques, laboratory tests (haematology, chemistry and urinalysis), physical examination and data collection of treatment-related toxicities. Tumour progression was defined as an increase of at least 25% in the overall area of the tumour size or the appearance of any new lesions. Deaths were also reckoned in progression. Time-related events were measured from the date of diagnosis.

The primary end point of this study was to compare progression-free survivals of the two cohorts. For this case, the two-tailed, unstratified log-rank test (Kaplan–Meier method), where *P*<0.05 indicates statistical significance, was used. For other comparisons, the *z*-test, Mann–Whitney's *z*-test, Fisher's exact test and Student's *t*-test were applied.

For the analysis of the effects of different variables (age, sex, disease staging, chemotherapy, radiotherapy, MSC administration) on survival, Cox's proportional hazards (PH) model was used. The Cox PH assumptions for this analysis have been evaluated for the six independent variables taken into account (see also Table 3) by using the Schoenfeld residuals (Schoenfeld, 1982) of the general form


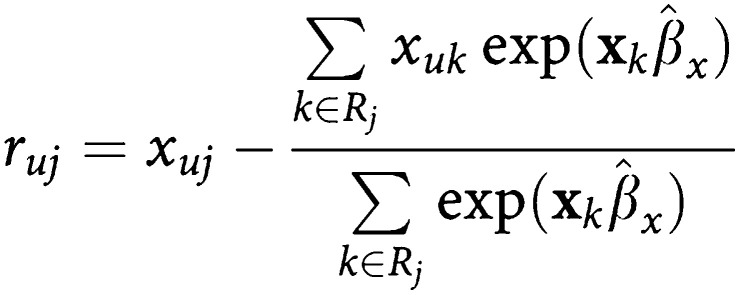


for each covariate *x*_*u*_, such that *r*_*uj*_ is the difference between the covariate value for the failed *j*th observation and the average of the covariate values, weighted on the basis of estimated hazard from the Cox model; the residual analysis carried out by means of the generalised method, developed by Grambsch and Therneau (1994), has shown no evidence of violations of Cox's PH assumptions.

## RESULTS

Between November 1998 and March 2001, 176 consecutive colorectal cancer patients from three oncosurgical institutions (at Uzsoki Teaching Hospital of Budapest, University of Szeged and University of Debrecen, Hungary) entered the study. Medix CRO Company independently collected data in the centres. The patients had either new diagnosis of their cancer or arrived for routine check-up of their previously diagnosed and treated disease. After progression of their disease, six patients of the control cohort started to take MSC on their own. These patients were not included in the data analysis.

Characteristics of the patients are shown in Table 1

**Table 1:** Baseline characteristics of the patients

	**MSC (*n*=66)**		**Control (*n*=104)**	
	**No.**	**%**	**No.**	**%**
Sex				
Male	40	60.6	57	54.8
Female	26	39.4	47	45.2

Age^a^ (years)				
Mean	61.7		66.1	
Range	36–88		40–79	
				
UICC classification of the disease^b^				
Stage I	5	7.6	20	19.2
Stage II	17	25.7	48	46.2
Stage III	26	39.4	32	30.8
Stage IV^c^	18	27.3	4	3.8
				
Recurrent disease^d^	4	6.1	1	1
				
Metastatic lesions				
Overall^e^	21		4	
Liver^f^	13		4	
Lung^g^	6		0	
Other organs^h^	2		0	
				
Time from diagnosis to present evaluation^h^ (months)				
Mean	29.6		34.0	
Median	23.5		28.0	
s.d.	15.8		20.2	
s.e.m.	1.9		2.0	
Range	7–71		7–61	
				
Chemotherapy, number of patients received^h^	43	65.2	53	51.0
				
Radiotherapy, number of patients received^i^	18	27.3	56	53.8
				
Length of MSC treatment (months)				
Mean	18.3			
Range	7–31			

a *P*=0.004.

b Mann–Whitney's *z*=4.618, *P*<0.001.

c *P*<0.001.

d No significant difference.

e Fisher's exact test: *P*<0.01.

f Fisher's exact test: *P*<0.01.

g Fisher's exact test: *P*<0.01.

h No significant difference.

i *z*=3.406, *P*<0.001.

. The two cohorts were obviously not balanced due to the fact that treatment assignment was directed by patients' preference. The average age of the patients in the control cohort was significantly higher than that of the MSC one (66.1 *vs* 61.7 years; *P*=0.004). In contrast, the MSC patients had significantly more advanced disease stages (Mann–Whitney probe: *z*=4.618; *P*<0.001). As many as 27.3% of the MSC patients already had stage IV of the disease (metastatic), while this value for the control patients was only 3.8 % (*P*<0.001). Moreover, the average length of time from diagnosis to the entry into the study was significantly longer for the MSC cohort than for the control cohort, 11.2 months and 1.1 months, respectively (*P*<0.001). There was no significant difference between the average length of time from diagnosis to the last visit completed between the two cohorts, 29.6 months and 34.0 months, respectively (Student's *t*=1.494; *P*=0.137). There was also no significant difference between the two cohorts in the number of patients receiving chemotherapy (*z*=1.819; *P*=0.069). However, significantly more patients of the control cohort had received radiotherapy (*z*=3.406; *P*<0.001). Except for mean age at entry, at baseline the prognoses of the MSC patients were significantly poorer than those of the control patients.

At end-point analysis, progression-related events (new recurrent disease, new metastatic lesions, deaths) were significantly more frequent in the control cohort (Table 2

**Table 2:** Occurrence of progression-related events (end-point analysis)

	**MSC (*n*=66)**		**Control (*n*=104)**	
	**No.**	**%**	**No.**	**%**
Patients with new recurrent disease^a^	2	3.0	18	17.3
Patients with new metastatic lesions^a^	5	7.6	24	23.1
Deaths^a^	8	12.1	33	31.7
Overall patients with progression events^b^	11	16.7	44	42.3

a *P*<0.01.

b *P*<0.001.

). Log-rank analyses (Kaplan–Meier estimates) also showed significant differences in favour of the MSC patients in the cumulative probabilities of both progression-free and overall survivals (Figures 1

**Figure 1:**
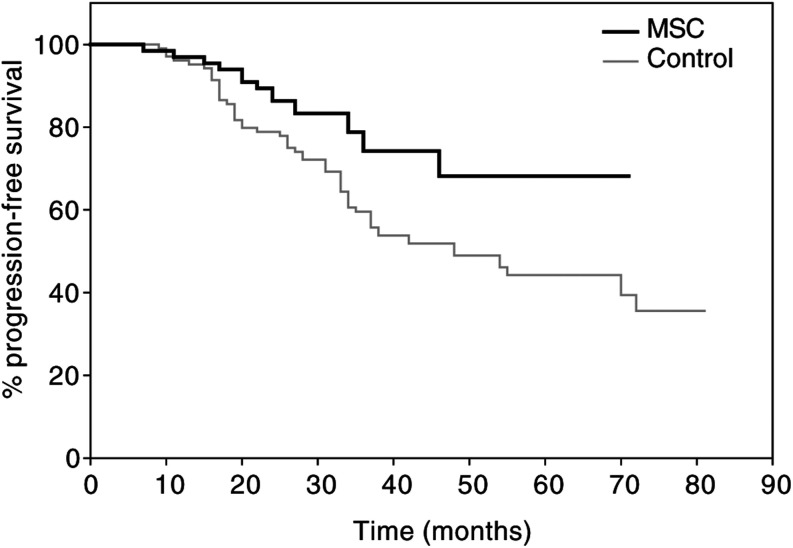
Kaplan–Meier estimate of the cumulative probability of remaining free from disease progression in colorectal cancer patients. Log-rank test: *χ*^2^=5.32; *P*=0.0184.

and 2

**Figure 2:**
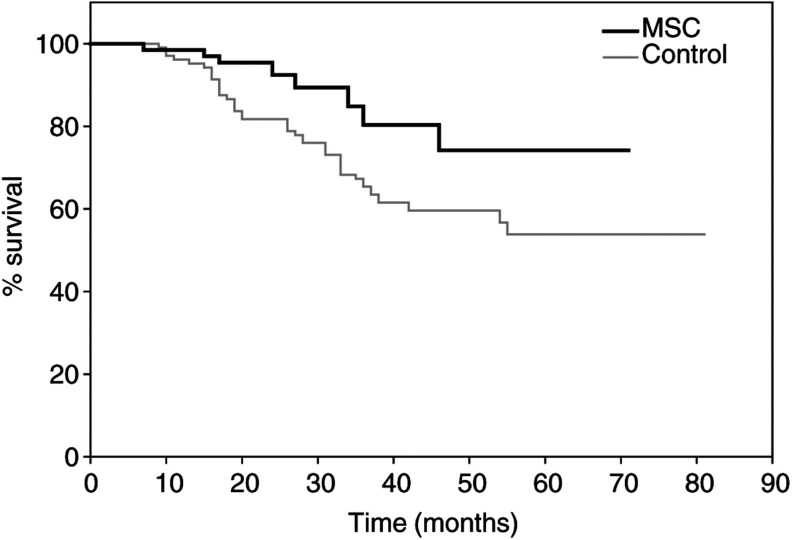
Kaplan–Meier estimate of the cumulative probability of overall survival in colorectal cancer patients. Log-rank test: *χ*^2^=4.66; *P*=0.0278.

).

Strong predictors of survival in a Cox's proportional hazards model (variable follow-up) were UICC stage and MSC treatment only (Table 3

**Table 3:** Multivariate analysis of survival of colorectal patients (Cox-regression), proportional hazards model: *χ*^2^=22.756; *P*=0.0009

**Variable**	***β***	**s.e.m.**	**Significance**	**Exp(*β*)**	**95% CI**
UICC staging	0.704	0.197	*P*=0.0004^a^	2.02	1.37–2.98
MSC treatment	−1.103	0.388	*P*=0.0045^a^	0.33	0.16–0.71
Age	0.236	0.015	*P*=0.113	1.02	0.99–1.05
Sex	0.261	0.276	*P*=0.342	1.30	0.76–2.23
Radiotherapy	−0.204	0.311	*P*=0.513	0.82	0.44–1.50
Chemotherapy	−0.088	0.336	*P*=0.794	0.92	0.47–1.77

a Denotes significant effect.

).

The administration of the medical nutriment was safe. No serious adverse event (NCI-CTC Grades 3–4) was observed. Generally, the greatest disadvantage of this preparation came from its rather unpleasant taste, but no violation of the compliance was detected, and according to blood analyses, no variation in the degree of absorption of MSC by the patients was found. With respect to the MSC cohort, the following side effects of MSC (with the absolute number of cases) were reported: diarrhoea (four), nausea and vomiting (two), flatulence (one), repletion (one), soft stool (one), constipation (one).

## DISCUSSION

As far as we know, the present study is the first one in the literature, which brings clinical data on the anticolorectal cancer treatments supporting activity of a fermented extract of one of the staple foods of mankind: wheat.

The study was carried out to analyse the results of the long-term supportive administration of MSC to colorectal cancer patients. There are shortcomings to this cohort trial design, which were accepted on ethical bases. As a result of the patients' preference-driven patient allocation, the two cohorts became unbalanced, but in favour of the control patients, regarding the prognostic factors (except mean age). The unbalanced cohorts did not compromise this data collection. Since the MSC cohort had worse prognostic factors at the time of recruitment, it was considered that any benefit detected over the control cohort could support the claim that MSC might be beneficial to colorectal cancer patients. It could be concluded that this wheat extract, in combination with surgery plus radio/chemotherapy, may inhibit overall tumour progression, including the formation of new metastases, and may prolong the survival of colorectal cancer patients. The Cox's regression analysis identified UICC staging and the more than 6 months long MSC administration as independent predictors of survival. The age of the patients enrolled had no significant influence. As MSC was given in combination and, for the majority (87%) of the radio/chemotherapy-treated patients, simultaneous with radio/chemotherapy, its effects could not be separated from the benefits of the conventional treatment modalities, nor could the possibly beneficial impact of MSC administration on previously applied radio/chemotherapy be measured. It is interesting that similar to its 70% neoplastic tumour-preventing effect in an experimental colon carcinogenesis model (Zalatnai *et al*, 2001), in the present study MSC decreased the risk of death among colorectal cancer patients by nearly 70%. (see the exp(*β*) values listed in Table 3, calculating the following: 100 × (1–0.33)=0.67=67%).

Besides these observed benefits of supplemental MSC in human colorectal cancer, it has recently been shown in a randomised trial that this extract is also beneficial concerning disease progression in stage III melanoma patients (Demidov *et al*, 2002). It has also been reported that MSC supplementation improves quality of life and alleviates fatigue in advanced lung cancer (Hidvégi *et al*, 2003).

MSC displays multiple effects on the immune system. The downregulation of MHC class I molecules on the membrane surface of the tumour cells by MSC increases the natural killer cell activity against neoplastic cells (Fajka-Boja *et al*, 2002). The upregulation of tumour necrosis factor alpha (TNF-*α*) secretion by the MSC-treated macrophages increases the antitumour activity of these cells (Telekes and Hidvégi, 2001). It is noteworthy that MSC enhances the activity of two of the most effective anticancer cellular immune mechanisms. It has also been shown that MSC upregulates the expression of intercellular adhesion molecule-1 (ICAM-1) on the endothelial cells (Telekes and Hidvégi, 2001). It is known that endothelial cells of the vasculature of human solid tumours have a decreased expression of ICAM-1 compared to normal endothelial cells' tissue, and this phenomenon can be considered as a tumour-derived escape mechanism since the development of an efficient leukcocyte infiltrate of the tumour is impaired (Griffioen *et al*, 1996). MSC not only induced the production of ICAM-1 but also showed synergy with the similar effect of TNF-*α*. These observations may, at least partly, explain the antimetastatic effect of MSC.

It has also been demonstrated that MSC induces apoptosis of cancer cells (Fajka-Boja *et al*, 2002). A recent study of the underlying mechanism behind the programmed cell death induction effect of MSC has pointed to the involvement of poly(ADP-ribose) polymerase cleavage and thus to the role of caspase-3, which indicated that decreasing cell motility and therefore inhibition of metastasis could also be direct consequences of MSC treatment (Comín-Anduix *et al*, 2002).

A further plausible hypothesis on the mechanism behind these positive clinical findings might come from an experiment in which MSC also showed significant therapeutic benefit in naive mice with lupus (SLE). The extract ameliorated the disease, downregulated the autoantibody production and increased Th1- and delayed Th2 responses (Ehrenfeld *et al*, 2001). The shifting of the Th1/Th2 balance towards an increased cellular immune response might have positive consequences in the mentioned human cancers.

Quite apart from its immunological effects, MSC has been shown to selectively and dose-dependently inhibit nucleic acid ribose synthesis in cancer cells through the regulation of enzymes involved in the nonoxidative steps of the pentose cycle, while increasing direct glucose carbon oxidation toward fatty acid synthesis (Boros *et al*, 2001, 2002; Cascante *et al*, 2002). Decreased substrate carbon flow through the pentose cycle toward nucleic acid synthesis in response to MSC treatment of cancer cells may also lead to a shortage of reducing equivalents that are indispensable for the reduction of ribonucleotides to deoxyribonucleotides during the replication of DNA (Comín-Anduix *et al*, 2002). The simultaneous decrease both in nucleic acid synthesis from glucose-derived ribose and in DNA replication may lead to a decrease in cancer cell proliferation, which may explain the slow disease progression and the increased survival rate in the MSC cohort.

It is likely that the combination of these mechanisms will provide enough theoretical basis to explain the supportive value of the fermented wheat germ extract in the treatment of colorectal cancer.
